# Prolonged grief: setting the research agenda

**DOI:** 10.3402/ejpt.v6.27303

**Published:** 2015-05-19

**Authors:** Rita Rosner

**Affiliations:** Department of Clinical and Biological Psychology, Catholic University Eichstätt-Ingolstadt, Eichstätt, Germany

**Keywords:** Bereavement, prolonged grief, complicated grief, treatment, dissemination

## Abstract

**Background:**

Prolonged grief disorder is proposed for the International Classification of Diseases (ICD-11), though it was rejected as a diagnosis for DSM-5.

**Objective:**

This review outlines findings and defines important areas for future research viewed from a lifespan perspective.

**Results:**

The development and psychometric evaluation of measures for the new diagnosis is paramount, specifically for children and adolescents. Treatments need to be adapted for specific subgroups and research findings have to be disseminated into various professional settings.

Abnormal forms of grief have been discussed for a very long time and a number of definitions formulated in the 1990s stimulated further research in this field. The terms “complicated grief” and “traumatic grief” (Horowitz et al., 1997; Prigerson et al., 1997; Shear, Zuckoff, & Frank, 2001) described similar syndromes, yet each term emphasises specific aspects: Whereas complicated grief highlights qualitative differences in abnormal grief, traumatic grief stresses one of the risk factors, which is the suddenness of the loss. “Prolonged grief disorder” (PGD), which is likely to be introduced in the International Classification of Diseases (ICD-11), stresses that prolonged grief, although similar to acute grief, lasts longer (Maercker et al., 2013). Core symptoms are intense yearning for and preoccupation with the deceased; reactive distress symptoms, such as feeling stunned or shocked by the loss; avoidance of reminders of the reality of the loss and emotional numbing; and finally social/identity disruption, such as feeling detached or having difficulties trusting others (Prigerson et al., 2009). As yearning for the deceased is the core symptom, a number of empirical studies have relied on attachment theory (Bowlby, 1980) and its successor, continuing bonds theory (Neimeyer, 2000). Other theories such as evolutionary perspectives, grief stage models, or cognitive stress theories have been discussed as well, but vary in terms of their empirical support (Stroebe, Hansson, Schut, & Stroebe, 2008; Stroebe, Schut, & Van den Bout, 2013). Experimental psychopathology studies currently focus on autobiographical memory and rumination, as well as biological factors. Important as this area is for the understanding of prolonged grief and the development of treatment models, a presentation of results would exceed the limits of this overview. Other interesting aspects such as predictors of PGD (Piper, Ogrodniczuk, Joyce, & Weidemann, 2011) and particularly those predictors specific to PGD (and not only to internalising disorders in general) also had to be omitted.

The same goes for the numerous biological aspects of PGD. In her extensive review, O'Connor (2012) recounts evidence for the notion of PGD as an attachment related disorder, over and above a general stress response. However, a detailed summary is beyond the limits of this paper.

Instead we will focus on diagnosis, measures, comorbidities, and treatment. To avoid confusion, the term PGD will be used throughout this paper.

Whereas the first part focuses on adults, the second part describes important issues concerning children and adolescents.

## Criteria in DSM and ICD

The aforementioned discussion about different diagnostic criteria has not yet been solved. Out of a reluctance to pathologize grief, which has been articulated both in scientific articles (Lilienfeld, 2007) and the popular press in general (Granek & O'Rourke, 2012), the authors of DSM-5 (American Psychiatric Association [APA], 2013) made three decisions affecting the way chronic and disabling grief will be handled in the future: They used a new combination of symptoms to define the syndrome, and termed it “persistent complex bereavement disorder.” This syndrome was relegated to the appendix for further study. At the same time the bereavement exclusion was removed from the diagnostic criteria of major depressive disorder. These changes may result in less optimal patient care: First they may lead to grieving patients being treated prematurely with antidepressants, which have not been shown to be particularly successful in improving prolonged grief symptoms (Bui, Nadal-Vicens, & Simon, 2012). Second as “persistent complex bereavement disorder” is only found in the appendix, and not considered a “real” diagnosis, sufferers may not be recognised at all or only treated for depression without receiving a grief-specific psychotherapy. Third, the only alternative, adjustment disorder, which would at least fit for some of the patients with prolonged grief, is defined by a duration of not more than 6 months “once the stressor or its consequences have terminated” (APA, 2013, p. 287). Furthermore, symptoms should not represent normal bereavement. Treatment studies reveal that time between the actual loss and start of treatment is several years on average (Papa, Sewell, Garrison-Diehn, & Rummel, 2013; Rosner, Pfoh, Kotoučová, & Hagl, 2014), which means adjustment disorder will not be a fitting diagnosis either. Details about pros and cons of DSM-5 decisions can be found in Wakefield (2013) and Bryant (2014), who conclude that “the DSM-5 decision is unlikely to have an impact on future research agendas” (p. 21).

Contrary to DSM-5, ICD-11 is likely to include PGD as a separate diagnosis amongst others in a new category for stress related disorders (Maercker et al., 2013). This will stimulate further research and will help clarify a number of important issues.

## Development of measures

Questionnaires and interview measures are needed based on the ICD-11 criteria with good psychometric properties. Currently the Inventory of Complicated Grief (Prigerson et al., 1995) is the most widely used questionnaire, however, different versions are used in studies. And although the overlap with the proposed ICD-11 criteria is large, there are still a number of smaller differences. In the area of interview measures the situation is more complex. None of the currently used and available measures has been used in a great number of studies, thus hindering the evaluation of results between studies.

Future research agenda: Develop and test reliable and valid interview measures.

## Consequences of valid criteria and measures

Differences in research methodology (amongst those the applied criteria, measures, and sample characteristics) lead to varying prevalence estimations in different countries. Although United States studies estimate PGD at around 10% (Mancini, Bonnano, & Clark, 2011), most other countries report smaller numbers: Switzerland 4.2% (Forstmeier & Maercker, 2007), Germany 3.7% (Kersting, Brähler, Glaesner, & Wagner, 2011), and Netherlands 4.8% (Newson, Boelen, Hek, Hofman, & Tiemeier, 2011). Each of the cited studies used different criteria and different measures. Apart from these methodological aspects, war-torn countries report much higher numbers, of course.

Furthermore, a broader range of comorbidities needs to be considered. Many studies report on the next diagnostic neighbours – major depression and PTSD (Maercker & Znoj, 2010), which cannot only be reliably differentiated from PGD (Boelen & Van den Bout, 2005), but are also often comorbid. Other comorbidities have not been frequently studied, even though we found surprisingly high comorbidities with pain (Rosner et al., 2014), and eating disorders (Rosner, Lumbeck, & Geissner, 2011). Comorbidities as these might get overlooked. Therefore we need more information on the complete comorbid spectrum and specifically so if they are related to age.

Future research agenda: Estimate prevalence rates and comorbidities based on the new criteria and measures.

## Treatment

Meta-analyses on grief treatments paint a clear picture: In general, studies on the efficacy of treatments for normal grief report small (Currier, Neimeyer, & Bermann, 2008: *d=*0.16; Fortner, 2000: *d*=0.13; Kato & Mann, 1999: *d*=0.11; Wittouck, Van Autreve, De Jaegere, Portzky, & Van Heeringen, 2011: *d=*0.03) to medium (Allumbaugh & Hoyt, 1999: *d*=0.43) effect sizes. Studies on patients with severe grief symptoms showed larger effect sizes than studies with subjects that did not have substantial grief symptoms: *d*=0.51 (Currier et al., 2008); *d*=0.53 (Wittouck et al., 2011). A number of clinical trials were published in the last years and showed large effect sizes for cognitive behavioural treatment protocols (Boelen, De Keijser, Van den Hout, & Van den Bout, 2007; Papa et al., 2013; Rosner et al., 2014; Shear, Frank, Houck, & Reynolds, 2005) in completer as well as in intent-to-treat analyses. Outcomes were stable in follow-up studies (Boelen, et al., 2007; Rosner, Bartl, Pfoh, Kotoučová, & Hagl, submitted).

## Elements of successful grief treatments

In an earlier meta-analysis (cited in Rosner, Kotouĉová, & Pfoh, 2011), we looked at the “ingredients” of successful interventions (defined as having ES > 0.80). Efficacious manuals included specific interventions to build rapport and enhance treatment motivation, to confront painful aspects, and to allow reconciliation and integration of the new and changed relationship to the bereaved. Shear (2015) reviewed the common elements in greater detail and mentioned similar aspects, though in different terms: Establishing lay of the land (psychoeducation), promoting self-regulation, building memories, setting aspirational goals, revisiting the world, storytelling, and using memory. Specifically the exposure to painful aspects of the loss has been studied in two trials. Boelen et al. (2007) compared two active versions of CBT vs. supportive counselling and reported an ES of *d=*1.36 for the combination of cognitive restructuring followed by exposure, and an ES of *d*=1.8 for the combination exposure followed by cognitive restructuring. Bryant et al. (2014) compared CBT with exposure therapy (CBT/exposure) or CBT alone and found the CBT/exposure condition to be superior to CBT alone. Thus exposure per se and exposure before cognitive restructurings seems to be most promising.

Summarising the above looking at therapeutic processes reveals similarities between efficacious interventions. Yet, in contrast to Shear (2015) who focused on communalities, I think that there is at least one trial studying a differing treatment concept. Papa et al. (2013) relied mostly on behavioural activation and reported considerable treatment success. Thus the question arises whether different approaches can be equally successful or if the efficacy is tied to sample characteristics.

Future research agenda: Explore different treatment approaches.

## Interventions tailored to specific groups

Taking a closer look at the above listed clinical trials suggests that there may be relevant subgroups of patients who either need an adaptation of treatment or a higher treatment dose. The death of one's child, for example, is a devastating experience (Bogensperger & Lueger-Schuster, 2014) that has been identified as a risk factor for the development of PGD and as a possible moderator of treatment outcome (Piper et al., 2011). Parents who have lost children drop out of treatment more often and achieve smaller treatment gains (Boelen, De Keijser, Van den Hout, & Van den Bout, 2011; Shear et al., 2005). Treatment adaptations should specifically address the needs of this group. Another group possibly requiring different approaches are those bereaved by suicide. One study showed especially large drop-out rates, which may be a hint to particular high avoidance within this group (Pfeffer, Jiang, Kakuma, Hwang, & Metsch, 2002). However, results are mixed and burdened by severe methodological problems (McDaid, Trowman, Golder, Hawton, & Sowden, 2008). A recent study on a CBT-based preventive psychoeducational intervention showed no significant effect on the development of complicated grief reactions, depression, and suicide risk factors among people bereaved by a suicide (Wittouck, Van Autreve, Portzky, & Van Heeringen, 2014). And finally, there are elderly patients. Clinical observations in our own treatment studies suggest they do not only suffer from PGD, but become increasingly lonely by multiple losses in their environment. Social contacts and behavioural activation may be good treatment strategies in this subgroup (cf., Papa et al., 2013).

Future research agenda: Adapt treatments to the needs of specific subgroups.

## Sequencing of treatment

Patients with PGD often show comorbid depression, PTSD, or substance abuse when they start treatment. As yet, there are no studies addressing treatment sequencing in these cases. One hypothesis could be that PTSD needs to be treated first, as avoidance may impede the treatment of PGD. Similar questions arise for comorbid substance abuse, pain, or depression.

Future research agenda: Test valid treatments and sequencing in case of specific comorbidities.

## Negative effects of treatment

Another point needs to be considered especially carefully in the area of PGD: There is some limited evidence that preventive or non-indicated treatment may produce harm, based on the results of meta-analyses showing very poor results for grief treatments in general, i.e., mostly normal grievers. Also, Fortner's (2000) much-discussed thesis reported negative effects for interventions in non-clinical mourners. Based on these publications and on a reluctance to pathologize grief, it is assumed that interventions for the bereaved may be harmful and that PGD is not a useful diagnosis. These discussions may have led to the critical attitude towards PGD in the USA. However, current randomised clinical studies on PGD patients do not report clinically significant deterioration in patients (Papa et al., 2013; Rosner et al., 2014). Nevertheless, given this history, future treatment studies should specifically focus on possible deterioration in treatment.

Future research agenda: Report specifically on deterioration in treatment.

## Pharmacological interventions

Studies concerning psychopharmacological interventions are scarce. Bui et al. (2012) summarise the available literature (three open-label trials, one randomised trial on bereavement-related depression, and four very small open label-trials of selective serotonin reuptake inhibitors) and report tentative support for the alleviation of depressive symptoms, rather than PGD symptoms. One study reports no positive effects for grief symptoms but more sleep problems instead in a diazepam intervention as compared to placebo (Warner, Metcalfe, & King, 2001). Given those very preliminary results, medication is as of yet probably only recommendable if patients suffer from comorbid depression. In combination with the equally sparse data base on specific biological correlates of PGD, it is even more necessary to study these to develop ideas about possible pharmacological interventions.

Future research agenda: Explore possible pharmacological interventions.

## Dissemination

The non-effectiveness of grief interventions for grievers without PGD and the lack of support for antidepressants are two central issues when looking at dissemination and implementation of research findings. Given the large numbers of self-help books, bereavement groups etc., there is certainly a widespread interest in grief and its alleviation. Thus it seems essential to spread state-of-the-art knowledge about PGD. As the field is even more diverse (bereavement counsellors, lay persons serving as self-help group leaders, members of the clergy) than in the area of other disorders, disseminating these facts to the wider public will be a challenge.

Future research agenda: Develop dissemination strategies for empirically supported results about PGD in various professional settings.

## Children and adolescents

The issues mentioned above are even truer for children and adolescents. Current diagnostic criteria (Cohen et al., 2010) lack empirical support and a developmental view (Kaplow, Layne, Pynoos, Cohen, & Lieberman, 2012). Accordingly, valid measures are missing. New instruments are promising but largely untested (Layne, Kaplow, & Pynoos, 2011; Spuij et al., 2012; Unterhitzenberger & Rosner, in press). Layne et al. (2011) suggest a new instrument that includes four domains: separation distress, reactive distress and behaviour, disruptions in personal and social identity, and preoccupation with the circumstances of the death. The domains are related to Layne's Multi-Dimensional-Grief-Model that incorporates many aspects from the varying theories (i.e., attachment theory and continuing bonds) and adds developmental aspects such as addressing the role of the caretaker as well as circumstance-related distress and traumatic aspects of the actual loss. Two meta-analyses studied the treatment success for children and adolescents (Currier, Holland, & Neimeyer, 2007; Rosner, Kruse, & Hagl, 2010). Similarly to adults, it seems that children not showing elevated distress levels 6 months after the loss should not be treated. Although some studies showed large improvements, few of those have been replicated and the methodological quality does not always allow valid conclusions.

Accordingly, recommendations for the dissemination of knowledge are more restricted. Similar to adults, bereaved children and adolescents without permanently elevated distress levels should not be treated. For those with elevated symptom scores a clear recommendation for one treatment approach is not yet warranted.

Future research agenda:

1. Adapt criteria of PGD in a developmentally sensitive way to children and adolescents and develop reliable and valid interview measures.
2. Extend the range of comorbidities studied and define trajectories for children and adolescents.
3. Conduct empirically sound treatment studies for children and adolescents with elevated PGD scores.

Summarising the issues raised above it becomes clear that as a first step diagnostic issues for adults, and specifically for young people, need to be addressed. Field trials and other representative studies based on the new criteria as well as a detailed recording of comorbidities are paramount. Trials have shown grief-specific treatments to be highly efficacious. Yet, the therapeutic needs of specific groups as well as the sequencing of treatments regarding comorbidities will be a huge challenge for the research field. Last but certainly not least, the implementation of scientific knowledge into clinical practice will need a longstanding and continuing effort.

## Supplementary Material

Prolonged grief: setting the research agenda

Prolonged grief: setting the research agenda

Prolonged grief: setting the research agenda

Prolonged grief: setting the research agenda
