# Supplementary material for: Prolonged grief: setting the research agenda
Source: Eur J Psychotraumatol. 2015 May 19;6:10.3402/ejpt.v6.27303. doi: 10.3402/ejpt.v6.27303 (PMC4439410; doi:10.3402/ejpt.v6.27303)
Supplement: Prolonged grief: setting the research agenda [file EJPT-6-27303-s001.pdf]

Deuil prolongé: Mettre en place un agenda de recherche

Rita Rosner

Contexte : Le trouble de deuil prolongé a été proposé pour la classification internationale des maladies (ICD -11), bien que rejeté comme un diagnostic par le DSM-5.
Objectif: Cette étude décrit les résultats et définit des domaines importants pour les futures recherches en s'intéressant aux effets visibles au cours de la vie.
Résultats : Le développement et l'évaluation psychométrique des mesures pour ce nouveau diagnostic sont primordiaux, en particulier pour les enfants et les adolescents. Les traitements doivent être adaptés à des sous-groupes spécifiques et les résultats de recherche doivent être diffusés dans divers milieux professionnels.

Mots-clés: deuil, deuil prolongé, deuil compliqué, traitement, diffusion

Citation: European Journal of Psychotraumatology 2015, **6**: 27303 - http://dx.doi.org/10.3402/ejpt.v6.27303
