# Supplementary material for: Prolonged grief: setting the research agenda
Source: Eur J Psychotraumatol. 2015 May 19;6:10.3402/ejpt.v6.27303. doi: 10.3402/ejpt.v6.27303 (PMC4439410; doi:10.3402/ejpt.v6.27303)
Supplement: Prolonged grief: setting the research agenda [file EJPT-6-27303-s002.pdf]

Elhúzódó gyász: Mit kutassunk?

Rita Rosner

Háttér: Az elhúzódó gyász a tervek szerint be fog kerülni a BNO-11-be, habár a DSM-5-be nem került be.
Célkitűzés: Az irodalmi áttekintés bemutatja az eddigi eredményeket és kijelöli a legfontosabb kutatási irányt minden életszakaszt figyelembe véve.
Eredmények: Az új diagnózis mérőeszközeinek fejlesztése és pszichometriai kiértékelése elsődleges különösen gyermekek és serdülők számára. A terápiákat adaptálni szükséges a speciális csoportok számára és a kutatási eredmények disszeminációja szükséges a klinikum különböző helyszíneire.

Kulcsszavak: veszteség; elhúzódó gyász; komplikált gyász; terápia; disszemináció

Citation: European Journal of Psychotraumatology 2015, **6**: 27303 - http://dx.doi.org/10.3402/ejpt.v6.27303
