# Supplementary material for: Prolonged grief: setting the research agenda
Source: Eur J Psychotraumatol. 2015 May 19;6:10.3402/ejpt.v6.27303. doi: 10.3402/ejpt.v6.27303 (PMC4439410; doi:10.3402/ejpt.v6.27303)
Supplement: Prolonged grief: setting the research agenda [file EJPT-6-27303-s003.pdf]

Duelo prolongado: Estableciendo el programa de investigación

Rita Rosner

Antecedentes: Se propone el trastorno de duelo prolongado para la clasificación internacional de enfermedades (CIE-11), aunque fue rechazado como diagnóstico por el DSM-5.
Objetivo**:** Esta revisión señala los resultados y define áreas importantes de investigaciones futuras vistas desde la perspectiva del ciclo vital.
Resultados: Es fundamental desarrollar y evaluar psicométricamente mediciones para el nuevo diagnóstico y específicamente para niños y adolescentes. El tratamiento ha de adaptarse a los diferentes subgrupos y se han de difundir los resultados en diversos entornos profesionales.

Palabras clave: duelo, duelo prolongado, duelo complicado, tratamiento, difusión

Citation: European Journal of Psychotraumatology 2015, **6**: 27303 - http://dx.doi.org/10.3402/ejpt.v6.27303
