# Supplementary material for: Prolonged grief: setting the research agenda
Source: Eur J Psychotraumatol. 2015 May 19;6:10.3402/ejpt.v6.27303. doi: 10.3402/ejpt.v6.27303 (PMC4439410; doi:10.3402/ejpt.v6.27303)
Supplement: Prolonged grief: setting the research agenda [file EJPT-6-27303-s004.pdf]

Uzun Süreli Yas: Araştırma Gündemi Oluşturma

Rita Rosner

Arkaplan: Hastalıkların Uluslararası Sınıflandırılması (ICD-11) için uzun süreli yas rahatsızlığı önerilmiş olsa da DSM-5 için tanı olarak reddedilmiştir.
Amaç: Bu yazı bulguların taslağını çıkarmakta ve yaşam süresi açısından ele alınan gelecek çalışmalar için önemli alanları tanımlamaktadır.
Sonuçlar: Ölçeklerin psikometrik değerlendirilmesi ve gelişimi yeni tanı, özellikle çocuklar ve ergenler için, oldukça önemlidir. Tedavilerin belirli alt gruplar için uyarlanmasına ihtiyaç duyulmakta ve araştırma bulguları çeşitli profesyonel alanlara yayılmalıdır.

Anahtar Kelimeler: yas, uzun süren yas, karmaşık yas, tedavi, yaygınlaşma

Citation: European Journal of Psychotraumatology 2015, **6**: 27303 - http://dx.doi.org/10.3402/ejpt.v6.27303
